# Supplementary material for: Insights into HP1a-Chromatin Interactions
Source: Cells. 2020 Aug 9;9(8):1866. doi: 10.3390/cells9081866 (PMC7465937; doi:10.3390/cells9081866)
Supplement: Supplementary file 1 [file cells-09-01866-s001.pdf]

cells

MDPI

Review

Insights into HP1a-Chromatin Interactions

Silvia Meyer-Nava, Victor E. Nieto-Caballero, Mario Zurita and Viviana Valadez-Graham *

Instituto de Biotecnología, Departamento de Genética del Desarrollo y Fisiología Molecular, Universidad Nacional Autónoma de México, Cuernavaca Morelos 62210, México; smeyer@ibt.unam.mx (S.M.-N.); vnieto@lcg.unam.mx (V.E.N.-C.); marioz@ibt.unam.mx (M.Z.)

* Correspondence: vvaladez@ibt.unam.mx; Tel.: +527773291631

Received: 26 June 2020; Accepted: 21 July 2020; Published: date

Supplementary Materials

Table S1. Publicly available ChIP-seq data for S2 cells.

Protein	Accession Number and Reference
dAdd1	GSE56101 [68]
H3K9ac	GSE20790 [256]
H3K9me3	GSE20794 [256]
HIPP1	GSE56101 [68]
HP1a	GSE56101 [68]
Su(var)3-7	GSE23487 [256]
Su(var)3-9	GSE27812 [256]
CP190	GSE41354 [257]
CTCF	GSE41354 [257]
Su(Hw)	GSE41354 [257]
Mod(mdg4)	GSE41354 [257]
GAF	GSE20770 [256]
Zw5	GSE32853 [16]
Ibf1	GSE46614 [190]
Ibf2	GSE46614 [190]

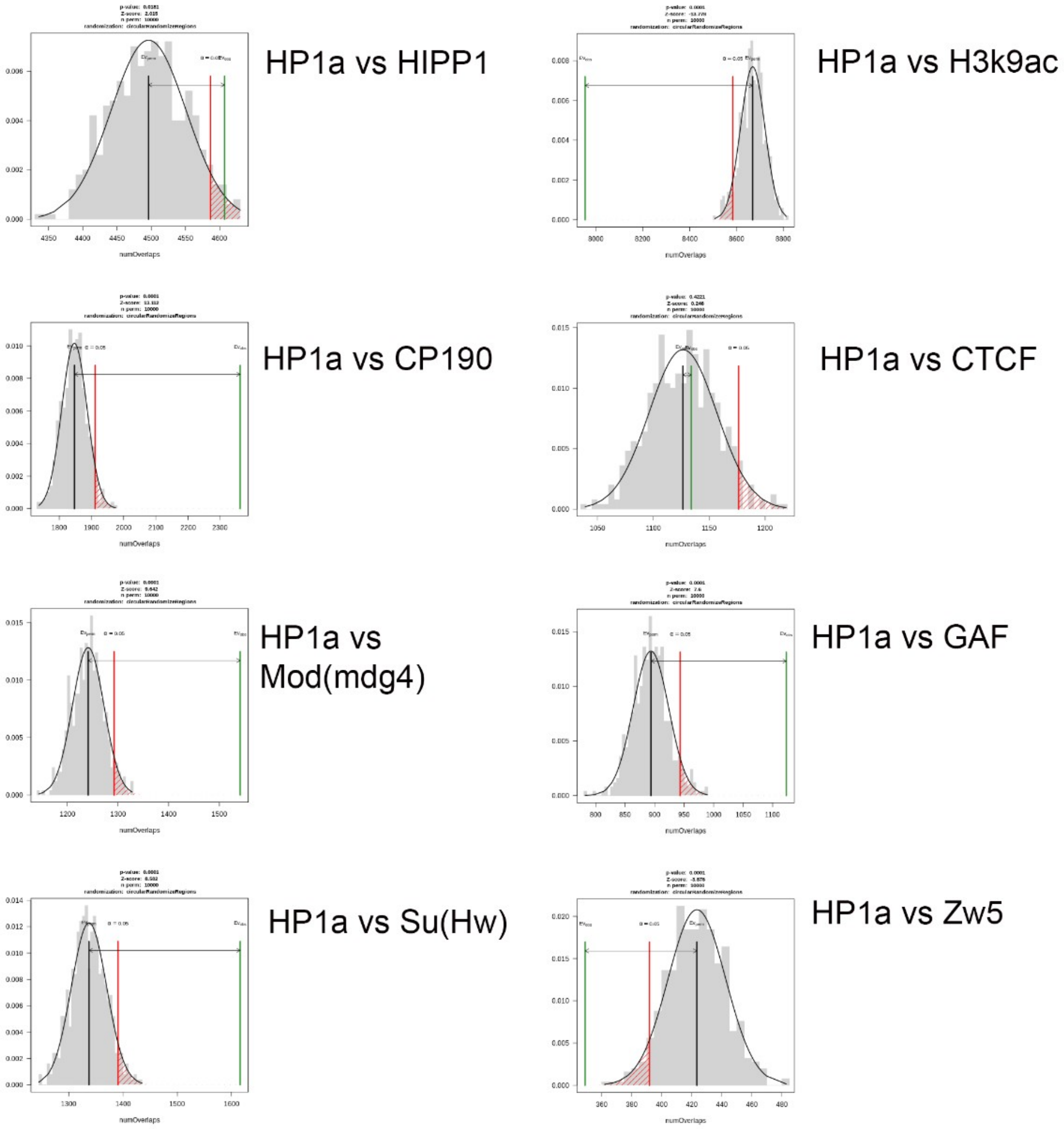

Figure S1. Permutation test for overlap between HP1a and architectural proteins. Permutation test-based R/Bioconductor package regioneR was used to assess whether peaks are significantly associated, based on $n = 10{,}000$ random permutations generated with the circular Randomize Regions algorithm. The gray histograms represent the number of overlaps of randomized set of regions associated with the indicated architectural protein. The black bar denotes the mean. In green is the mean overlap of the regions bound by HP1a with the tested regions (architectural proteins). The red bar denotes the significance limit set at $p < 0.05$. z-score measures the strength of the overlap irrespective of the number of permutations. High z-score and low p value indicate significant association. The data was significant with 95% confidence interval in the case of HIPP1 (p-value 0.0181), Su(Hw) (p-value 0.0001), CP190 (p-value 0.0001), Mod(mdg4) (p-value 0.0001), and GAF (p-value 0.0001). For CTCF (p-value 0.4221) and Zw5 (p-value 0.0001, z-score -3.876) data were not significant. H3K9ac was used as a negative control.

© 2020 by the authors. Licensee MDPI, Basel, Switzerland. This article is an open access article distributed under the terms and conditions of the Creative Commons Attribution (CC BY) license (http://creativecommons.org/licenses/by/4.0/).
